# Persisting Type 2 Endoleaks Following EVAR for AAA Are Associated With AAA Expansion

**DOI:** 10.1177/15266028221081079

**Published:** 2022-03-03

**Authors:** Johannes Hatzl, Vivian Wang, Maani Hakimi, Christian Uhl, Fabian Rengier, Thomas Bruckner, Dittmar Böckler

**Affiliations:** 1 Department of Vascular and Endovascular Surgery, University Hospital Heidelberg, Heidelberg, Germany; 2 Department of Vascular Surgery, Luzerner Kantonsspital, Luzern, Switzerland; 3 Clinic for Diagnostic and Interventional Radiology, University Hospital Heidelberg, Heidelberg, Germany; 4 Institute of Medical Biometry and Informatics, Heidelberg University, Heidelberg, Germany

**Keywords:** aorta, endoleak, EVAR, type 2 endoleak, persisting endoleak, AAA expansion

## Abstract

**Purpose:**

To determine the evolution of abdominal aortic aneurysm (AAA) diameter in the presence of persisting type 2 endoleaks (pEL2) following endovascular abdominal aortic aneurysm repair (EVAR).

**Materials and Methods:**

This is a retrospective, single-center, case-control study. All patients with pEL2 (pEL2 group, persisting for > 12 months) between 2004 and 2018 were identified and compared with a 1:1 age- and gender-matched control with no endoleak (control group). Primary outcome measures were freedom from AAA expansion and freedom from AAA shrinkage over time. AAA diameter measurements were performed on computed tomography angiography (CTA). Secondary outcome measures were survival, AAA-related mortality, reinterventions for pEL2, incidence of secondary type 1 endoleaks (EL1), and infrarenal aortic branch vessel anatomy.

**Results:**

A total of 773 patients were treated with EVAR for AAA between 2004 and 2018. Of them, 286 patients demonstrated type 2 endoleaks (EL2) in postoperative CTA or intraoperative angiography (37%). Forty-five of 286 EL2 (15.7%) were pEL2 (pEL2 group). Freedom from AAA expansion in the pEL2 group was 100%, 96.7%, 85.2%, and 54.3% after 1, 2, 3, and 4 years, respectively, compared with 100% after 1, 2, 3, and 4 years in the control group (p<0.01). Freedom from AAA shrinkage in the pEL2 group after 1, 2, 3, and 4 years was 95.5%, 90.4%, 90.4%, and 79.1%, respectively, compared with 86.7%, 34.8%, 19.3%, and 19.3% in the control group (p<0.01). Overall survival at 1, 2, 3, and 4 years was 100%, 97.6%, 95.0% and 95.0% in the pEL2 group and 100% at 1, 2, 3, and 4 years in the control group (p=0.17). There were no AAA-related deaths in either group. Patients with pEL2 had a significantly increased number of infrarenal aortic branches (p<0.05, respectively). Eighteen patients (40.0%) in the pEL2 group underwent 34 reinterventions for pEL2, with a median follow-up (FU) of 925 days (0–4173). Clinical success was achieved in 9 patients (50.0%). Four patients (8.9%) with pEL2 developed secondary EL1 after a median FU of 1278 days (662–2121).

**Conclusion:**

pEL2 are associated with AAA expansion during midterm FU. Further studies are warranted to evaluate the association of AAA expansion due to pEL2 with clinical outcomes to allow recommendations with regard to treatment indications.

## Introduction

Since its introduction by Parodi et al in 1991, endovascular abdominal aortic aneurysm (AAA) repair (EVAR) has expanded the treatment options of AAAs by improving perioperative and short-term outcomes when compared with conventional open aortic repair (OAR).^1^ However, a recent meta-analysis has demonstrated that EVAR is associated with an increased risk of AAA-related mortality, reintervention, and rupture after 8 years of follow-up (FU).^2^ Both, continued AAA expansion and absence of AAA shrinkage, have been identified as risk factors for long-term mortality following EVAR.^3,4^ Risk factors that have been associated with AAA expansion are age, gender, AAA diameter, AAA neck angulation, and AAA neck diameter as well as endoleak type 1 (EL1) and type 3 (EL3).^4,5^ The role of type 2 endoleaks (EL2) remains more controversial.^6–10^ EL2 are detected in about 25% of EVAR cases and are defined as retrograde flow into the AAA from aortic side branches, most commonly the lumbar arteries (LAs) and the inferior mesenteric artery (IMA) but also the median sacral artery (MSA) or accessory renal arteries.^7,11–15^ About half of these EL2 resolve spontaneously within 6 to 12 months and are therefore usually considered as a transient and benign phenomenon. Nonetheless, some EL2 persist over time and are associated with AAA expansion and even secondary AAA ruptures in some cases.^12,13,16–18^

This study aimed to determine the evolution of AAA diameter in patients with EL2 persisting for >12 months (persisting type 2 endoleaks [pEL2]) from a cohort of patients treated with EVAR over a period of 15 years in a single center.

## Materials and Methods

### Study Design

This is a single-center, retrospective, case-control study. Patients with pEL2 following standard EVAR for AAA were compared with an age- and gender-matched control group without EL. Primary outcome measures were freedom from AAA expansion and freedom from AAA shrinkage. Secondary outcome measures were overall survival, AAA-related mortality, outcomes of reinterventions for pEL2, incidence of secondary type 1 endoleaks (EL1), and infrarenal aortic branch vessel anatomy.

### Study Population and Imaging Material

All consecutive patients treated with EVAR for asymptomatic AAA between January 1, 2004 and December 31, 2018, were identified using a relational, clinical database. All available computed tomography angiography (CTA) scans of patients with pEL2 were included. Postoperative CTA was performed before discharge, at 1 year, and yearly thereafter. Based on individual assessment, intervals for CTA FU could be extended to 2 or more years if there were no signs of adverse findings in previous imaging. Instead, duplex sonography was used as imaging modality in these visits. The CTAs that demonstrated concomitant ELs of other types were excluded for the analysis of the primary outcome measure. The control group consisted of a 1:1 age- and gender-matched patient cohort without EL of any type and was randomly selected from the remaining patient pool. To be considered for the control group, all patients had to have at least 2 postoperative CTA scans available with a time difference of at least 12 months in analogy to the study group. Diagnosis of EL was carried out independently by a consultant radiologist and a qualified vascular surgeon based on CTA assessment. The diagnosis of EL2 was established based on postoperative CTA, which included native, arterial, and delayed phases (CTA). EL2 was defined according to generally recognized standards as retrograde flow from infrarenal aortic branch vessels without attachment site connection. Early and late EL2 were included. Early EL2 were detected ≤30 days following the index procedure. EL2 detected beyond 30 days were defined as late EL2.^19^ Basic demographic data, procedural data, and imaging data were added into the database model. Ethical approval was provided by the institutional review board and informed consent was obtained from all patients (S-160/2019).

### Imaging Assessment

**Figure 1.**
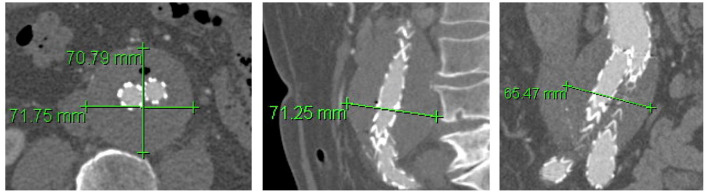
Measurements of maximum abdominal aortic aneurysm (AAA) diameter (outer-outer diameter) were performed in 4 planes in all available computed tomography angiography (CTA) scans (axial transverse, axial anterior-posterior, sagittal, and frontal; sagittal and frontal plane measurements were performed perpendicular to central lumen line).

Measurements of maximum aortic diameter (outer-to-outer diameter) were performed in 4 planes in all available CTA scans (axial transverse, axial anterior-posterior, sagittal, and coronal; diameters on sagittal and coronal planes were measured perpendicular to the lumen centerline; Figure 1). AAA sac expansion was defined as at least 5 mm increase in diameter in at least 2 of the 4 planes compared with the baseline postoperative CTA. Correspondingly, AAA sac shrinkage was defined as at least 5 mm decrease in diameter in at least 2 of the 4 planes. A stable AAA was defined as an AAA that did not demonstrate either AAA expansion or shrinkage according to this definition. Intraobserver and interobserver reliability of diameter measurements was evaluated by repeated measurements in 10 randomly selected patients by 2 readers and by 1 reader twice, respectively, and in random order. CTA scans had a slice thickness of equal to or less than 3 mm. The number of patent LAs and the patency of the IMA and the MSA were assessed and the maximum diameter of the largest LA artery as well as the diameter of the IMA were measured on axial slices. Persisting EL2 were subclassified as persisting endoleak type 2a (pEL2a) with 1 feeding vessel or persisting endoleak type 2b (pEL2b) with at least 2 feeding vessels leading to the same endoleak cavity. LA with a common stem were counted as 1 LA. Persisting EL2 with 2 feeding vessels leading to 2 separate cavities were classified as pEL2a. Clinical success following reintervention for pEL2 was defined as a technically successful procedure (no evidence of flow entering the aneurysm sac at the end of the procedure) that showed sac stability or a decrease in sac size on FU imaging, with or without residual endoleak.

### Statistical Design

Freedom from AAA expansion and freedom from AAA shrinkage as well as overall survival were visualized using Kaplan–Maier estimates and tested for statistically significant differences between groups using the log-rank test. In addition, 95% confidence intervals and standard errors are reported. The study end date was December 31, 2018. Based on this date, the follow-up index (FUI) was calculated.^20^ Intraobserver and interobserver reliability was tested using Kendall’s coefficient of concordance. Welch 2-sample *t* test was used to test for statistically significant differences of age, preoperative estimated glomerular filtration rates, preoperative AAA morphology characteristics, diameters of the IMA and LAs, and the maximum available FU. Fisher exact test for Count Data was used to test for statistically significant differences in gender distribution between groups. Furthermore, χ² test was used to test for significant differences between observed AAA shrinkage, AAA expansion, and AAA stability as well as IMA and MSA patency rates between groups. Matching of groups was performed using SQL and SAS software (SAS Institute, 2011). Statistical analysis was performed using “R-statistics.” Statistical significance was defined as α<0.05.

## Results

### Study Population

Between January 1, 2004 and December 31, 2018, 773 patients were treated with EVAR for AAA. Of them, 286 patients showed any EL2 in postoperative CTA or intraoperative angiography (37%). A total of 45 of 286 patients (15.7%) demonstrated an EL2 continuously persisting for more than 12 months in 2 postoperative CTA scans and thereby qualified for the pEL2 group (pEL2). These 45 patients with pEL2 had 137 CTA scans available (mean=3.0 CTs/patient) for analysis with a mean maximum FU of 962.4 days (SD=720.6). The corresponding 45 patients from the age- and gender-matched control group had 120 CTA scans (mean=2.7 CTs/patient) with a mean maximum FU of 962.1 days (SD=529.4). Per definition, the control group did not have any EL in any postoperative CTA. There was no statistically significant difference between the maximum FU time of available CTA scans (p=0.99). Both groups were comparable regarding age, gender, and preoperative renal function.

### Aneurysm Morphology and Infrarenal Aortic Branch Anatomy

Of the 45 patients with pEL2, 40 preoperative CTAs in the pEL2 group were available. Correspondingly, 42 CTAs of the 45 patients in the control group were available for assessment of preoperative aneurysm morphology and infrarenal aortic branch anatomy. Patients in the pEL2 group had a median of 4 (1–8) infrarenal LA. In the control group, the median number of LA was 3 (0–8; p<0.01). In the group of patients with pEL2, 3 (7.5%) patients had 2 or less LAs, 27 patients (67.5%) had 3 to 5 LAs, and 10 patients (25%) had 6 to 8 LAs. In the group of controls, 20 (47.6%) had 2 or less LAs, 16 (35.7%) had 3 to 5 LAs, and 7 (16.7%) had 6 to 8 LAs. The distribution of the number of LAs is illustrated in Supplemental Table 1 and Supplemental Figure 1.

Thirty-three patients (82.5%) in the pEL2 group had a patent IMA compared with 25 (59.5%) in the control group (p=0.04). In the pEL2 group, 20 patients (50%) had a patent MSA compared with 9 patients (21.4%) in the group of controls (p=0.013). The median diameter of the largest patent LA and the IMA was 2.3 mm and 2.6 mm in both groups. (p=0.46 and 0.43, respectively). The median maximum AAA diameter in the pEL2 group was 55 mm (38–78) and the median AAA neck length was 35 mm (17–65) with a AAA neck diameter of 24 mm (16–32) and a neck angulation of 35° (5–97). The control group had a median maximum AAA diameter of 55 mm (46–87) with a median neck length of 32 mm (10–45), a median neck diameter of 25 mm (18–32), and an angulation of 36° (7–83). There were no statistically significant differences in preoperative AAA morphological characteristics between groups. Demographics, comorbidity, AAA morphology, and infrarenal aortic branch anatomy are presented in Table 1. Histograms of the distribution of age and AAA morphology characteristics are presented in Supplemental Figures 2–6.

**Table 1.** Baseline Demographics, Comorbidity, Aneurysm Morphology, Infrarenal Aortic Branch Anatomy, and pEL2 Morphology.

	pEL 2 group	Control group	p value
N	45	45	
Demographics and comorbidity			
Age	73 (60–84)	76 (58–88)	0.22^a^
Male	43	43	1.0^b^
ASA 1	0	0	
ASA 2	15	10	
ASA 3	27	33	
ASA 4	3	2	
ASA 5	0	0	
Egfr	69.2 (6.2–100.5)	82.4 (49.7–101.4)	0.07^a^
AAA morphology			
AAA diameter	55 (38–78)	55 (46–87)	0.18^a^
AAA neck length	35 (17–65)	32 (10–45)	0.7^a^
AAA neck diameter	24 (16–32)	25 (18–32)	0.65^a^
AAA neck angulation	35 (5–97)	36 (7–83)	0.36^a^
Infrarenal aortic branches			
Number of patent Las	4 (1–8)	3 (0–8)	0.0005^a^
Number of patent IMAs	33 (82.5%)	25 (59.5%)	0.04^b^
Number of patent MSAs	20 (50.0%)	9 (21.4%)	0.013^b^
Maximum diameter Las	2.3 (1.4–3.5)	2.3 (1.4–3.2)	0.46^a^
Maximum diameter IMA	2.6 (1.6–4.3)	2.6 (1.0–4.3)	0.43^a^
Persisting EL2 morphology			
Early EL2	34 (75.6%)		
Late EL2	11 (24.4%)		
EL2a	24 (53.3%)		
Single cavity: LA	12 (50.0%)		
Single cavity: IMA	8 (33.3%)		
Two cavities: LA, IMA	3 (12.5%)		
Two cavities: IMA, acc. renal	1 (4.2%)		
EL2b	19 (42.2%)		
LA-LA	10 (52.6%)		
IMA-LA	8 (42.1%)		
IMA-acc. renal	1 (5.3%)		
Other	2 (4.5%)		

Medians with range and absolute and relative frequencies are presented; Lumbar arteries with a common stem are counted as 1 lumbar artery.

Abbreviations: ASA, American Society of Anesthesiologists; Early EL2, detected ≤30 days following the index procedure; eGFR, estimated glomerular filtration rate; IMA, inferior mesenteric artery; LA, lumbar artery; Late EL2, detected >30 days following the index procedure; MSA, median sacral artery; acc. renal, accessory renal artery.

a Welch 2-sample *t* test.

b χ² test.

### Type 2 Endoleak Morphology

Of the 45 patients with pEL2, 24 patients (53.3%) had a pEL2a and 19 patients (42.2%) had a pEL2b. Two pEL2 fulfilled the diagnostic criteria for an EL2 but the feeding vessel(s) could not be exactly determined (4.5%). Twenty of the patients with a pEL2a had a single cavity EL2 either through a LA (N=12, 50%) or the IMA (N=8, 33.3%). Four patients had a pEL2a with multiple cavities (N=4, 16.7%). Of the 19 patients with a pEL2b, 10 patients (52.6%) had 2 LAs feeding the cavity and 8 patients (42.1%) had the IMA and 1 LA feeding the endoleak cavity. Thirty-four patients had early EL2 (75.6%) and 11 patients had late EL2 (24.4%).

### Freedom From AAA Expansion

**Figure 2.**
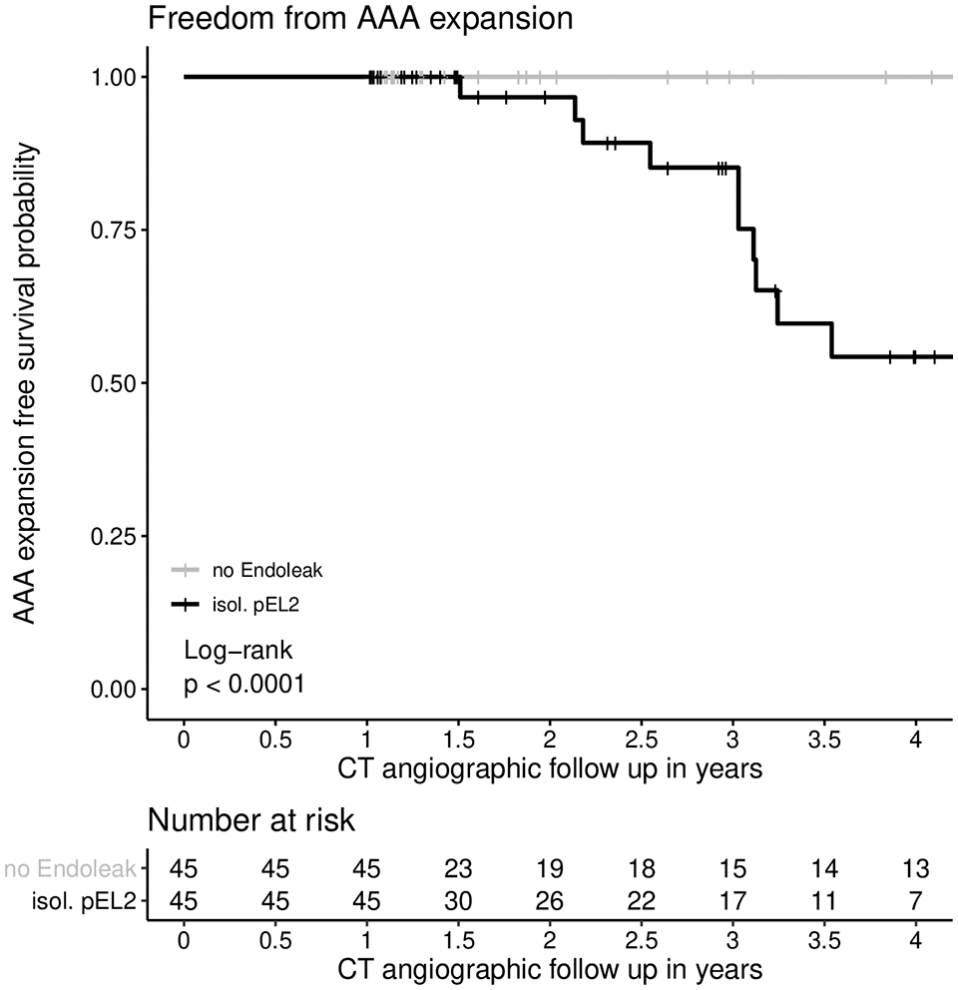
Freedom from AAA expansion. AAA expansion was defined as AAA diameter increase of at least 5 mm in at least 2 different planes. There was a statistically significant difference in freedom from AAA expansion between the control group without EL and the group of patients with isolated EL2 persisting for >12 months (p<0.01; log rank test was applied). Freedom from AAA expansion at 1 year in the pEL2 group: 100% (CI=100%–100%, SE=0), at 2 years: 96.7% (CI=90.5%–100%, SE=0.03), at 3 years: 85.2% (CI=72.7%–99.8%, SE=0.07), and at 4 years: 54.3% (CI=36.4%–80.8%, SE=0.11); Freedom from AAA expansion at 1, 2, 3, and 4 years in the control group: 100% (CI=100%–100%, SE=0). CI, confidence interval; AAA, abdominal aortic aneurysm; EL, endoleak.

Estimated freedom from AAA expansion in the pEL2 group was 100% (100%–100%), 96.7% (90.5%–100%), 85.2% (72.7%–99.8%), and 54.3% (36.4%–80.8%) after 1, 2, 3, and 4 years, respectively, of FU. In the control group without any EL, freedom from AAA expansion was 100% (100%–100%) after 1, 2, 3, and 4 years (p<0.01). Freedom from AAA expansion is presented in Figure 2.

### Freedom From AAA Shrinkage

**Figure 3.**
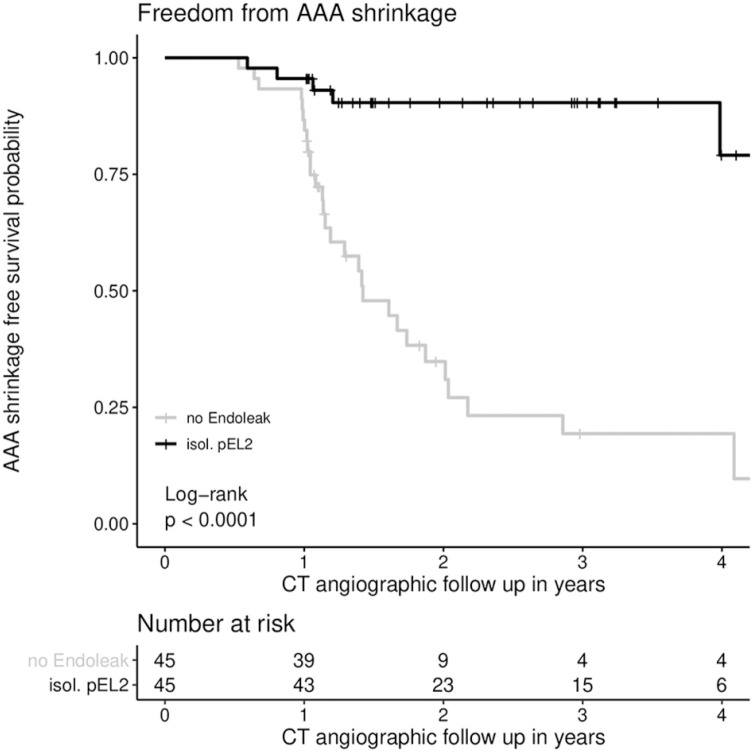
Freedom from AAA shrinkage. AAA shrinkage was defined as AAA diameter decrease of at least 5 mm in at least 2 different planes. There was a statistically significant difference in freedom from AAA shrinkage between the control group without EL and the group of patients with isolated, pEL2 persisting for >12 months (p<0.01; log rank test was applied). Freedom from AAA shrinkage at 1 year in the pEL2 group: 95.6% (CI=89.7%–100%, SE=0.03), at 2 years: 90.4% (CI=81.8%–99.9%, SE=0.05), at 3 years: 90.4% (CI=81.8%–99.9%, SE=0.05), and at 4 years: 79.1% (CI=58.8%–100%, SE=0.11); Freedom from AAA shrinkage at 1 year in the control group: 86.7% (CI=77.3%–97.2%, SE=0.05), at 2 years: 34.8% (CI=21.9%–55.5%, SE=0.08), at 3 years: 19.3% (CI=9.2%–40.8%, SE=0.07), and at 4 years: 19.3% (CI=9.2%–40.8%, SE=0.07). AAA, abdominal aortic aneurysm; EL, endoleak; CI, confidence interval.

Estimated freedom from AAA shrinkage in the pEL2 group after 1, 2, 3, and 4 years was 95.5% (89.7%–100%), 90.4% (91.8%–100%), 90.4% (81.8%–100%), and 79.1% (59.8%–100%), respectively. In the control group, freedom from AAA shrinkage was 86.7% (77.3%–92.2%), 34.8% (21.9%–55.5%), 19.3% (9.2%–40.8%), and 19.3% (9.2–40.8%) after 1, 2, 3, and 4 years, respectively (p<0.01). Freedom from AAA shrinkage is presented in Figure 3.

### AAA Expansion and AAA Shrinkage

Over the entire observation period, 15 patients (33.3%) from the pEL2 group (mean FU period: 962.4 days, SD=720.6) demonstrated AAA expansion, 25 patients had stable AAA diameters (55.6%), and 5 patients had a shrinking aneurysm (11.1%). In the group of controls (mean FU period: 962.1 days, SD=529.4) without EL, no patient experienced AAA expansion ≥5 mm in ≥2 planes, whereas 32 patients (71.1%) had AAA shrinkage of ≥5 mm in ≥2 planes (p<.01). There were no cases of patients who showed postoperative AAA expansion ≥5 mm in ≥2 planes at one point and shrinkage of the AAA ≥5 mm in ≥2 planes in a later CTA and vice versa. Proportions of patients with observations of AAA expansion and shrinkage are shown in Table 2.

**Table 2.** Proportions of Patients With Observations of AAA Shrinkage and Expansion.

	AAA shrinkage^a^	AAA expansion^b^	AAA stable	Mean FU (SD)
Control (N=45)	32 (71.1%)	0 (0%)	13 (28.9%)	962.1 days (529.4)
pEL2 (N=45)	5 (11.1%)	15 (33.3%)	25 (55.6%)	962.4 days (720.6)

Categorical data are presented as absolute and relative frequencies. Continuous data are presented as means with standard deviation.

Abbreviations: AAA, abdominal aortic aneurysm; FU, follow-up; pEL2, persisting type 2 endoleak.

a AAA shrinkage is defined as shrinkage of diameter ≥5 mm in at least 2 planes.

b AAA expansion is defined as expansion of diameter ≥5 mm in at least 2 planes (p<.0001; χ² test was applied).

There was a high intraobservership and interobservership concordance of measurements (Kendall’s coefficient of concordance=.99).

### Survival

**Figure 4.**
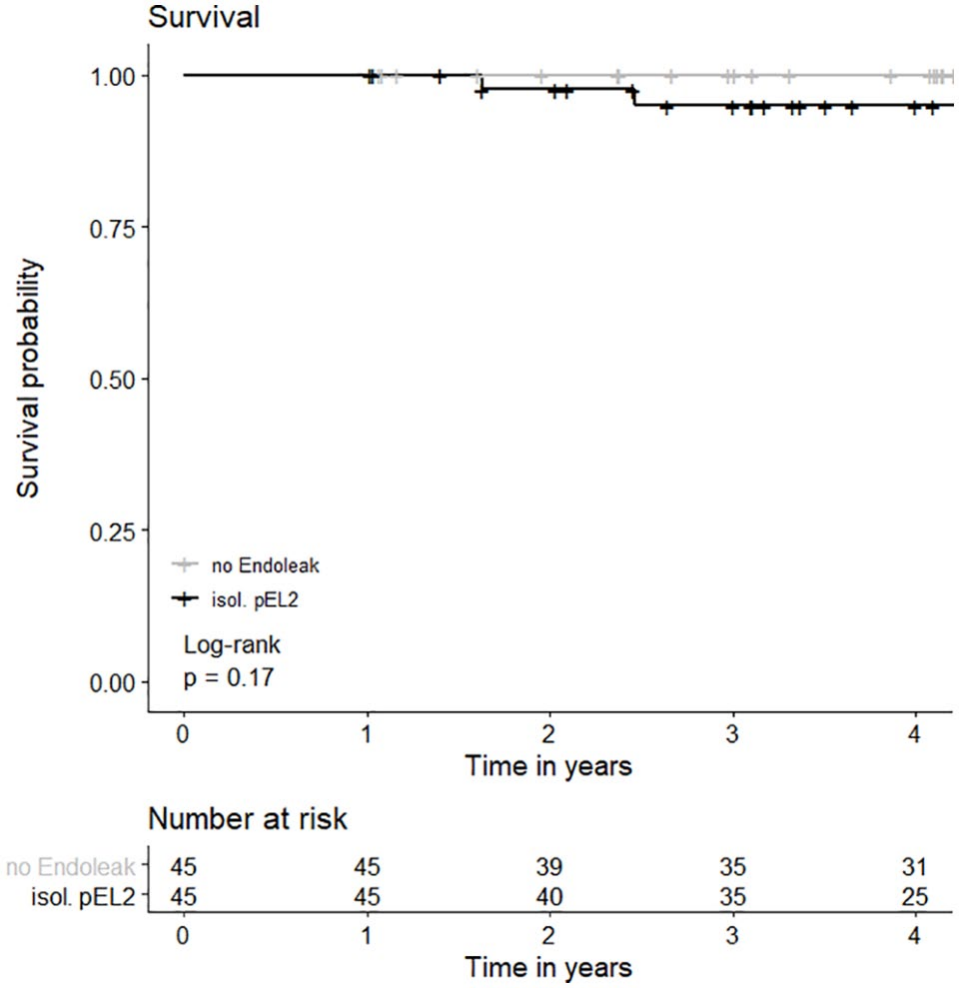
Overall survival. There was no statistically significant difference in overall survival between the control group without EL and the group of patients with isolated EL2 persisting for >12 months (p=0.17, log rank test was applied). Survival at 1 year in the pEL2 group: 100% (CI=100%–100%, SE=0), at 2 years: 97.6% (CI=93.1%–100%, SE=0.02), at 3 years: 95.0% (CI=88.4%–100%, SE=0.04), and at 4 years: 95.0% (CI=88.4%–100%, SE=0.35); Survival at 1, 2, 3, and 4 years in the control group: 100% (CI=100%–100%, SE=0). EL, endoleak; CI, confidence interval.

Estimated survival in the pEL2 group after 1, 2, 3, and 4 years was 100% (100%–100%), 97.6% (93.1%–100%), 95.0% (88.4%–100%), and 95.0% (88.4%–100%). In the control group, estimated survival was 100% (100%–100%) at 1, 2, 3, and 4 years (p=0.17). Survival is presented in Figure 4. There were no AAA-related deaths and no secondary AAA ruptures in either group. Over the entire observation period, in the pEL2 group, there were 6 recorded deaths. In 3 patients, the cause of death was unknown and 2 patients died from multi-organ failure following acute pancreatitis or peritonitis from a perforated gastric ulcer, respectively. One patient died from hemorrhagic shock following ruptured aortic dissection (Stanford B) 3510 days following EVAR. The median time to death was 1948 days (595–3781). In the group of controls, there was one recorded death, which had an unknown cause and occurred 2344 days following the index procedure. The FUI (SD) for the pEL2 group was 0.77 (SD=0.29) and in the control group it was 0.81 (SD=0.28).

### Reinterventions for pEL2

Eighteen of 45 patients (40.0%) in the pEL2 group underwent 34 reinterventions for pEL2. Indication for reintervention was AAA sac expansion (>5 mm) in all patients (100%). Reinterventions were performed after a median FU of 1336 days (249–3079). Nine of the pEL2 were preoperatively classified as pEL2a (50.0%) and 9 were classified as pEL2b (50.0%). The median FU following reintervention for pEL2 was 925 days (0–4173). Of the 18 patients, 9 patients underwent 2 or more procedures to treat the pEL2 (50.0%). Overall, clinical success, according to the abovementioned definition, was achieved in 9 patients (50.0%). Three patients (16.7%) were lost to FU following reintervention, so that no determination with regard to clinical success could be made. One patient is currently awaiting his next FU visit; until then, no determination of clinical success can be made. In one patient, during the intended intervention to treat a pEL2, a concomitant EL1a was detected. In one other patient, angiography for intended pEL2 treatment revealed an EL1b and no sign of EL2, therefore this patient was preoperatively and CT-angiographically misclassified as pEL2.

Two patients with clinically unsuccessful reinterventions to treat the pEL2 with slow progression were unfit for open surgical conversion due to comorbidity and the pEL2 was therefore managed conservatively. One patient had a clinically unsuccessful reintervention and was then lost to FU before a second reintervention could be performed. With regard to morbidity, 2 patients suffered from ischemia of the lumbosacral plexus following reinterventions for pEL2 (11.1%). In one patient, symptoms resolved spontaneously; the other patient remained wheelchair-bound at his last FU visit 71 days following the last reintervention for pEL2. One other patient had a false aneurysm at the femoral puncture site, which had to be repaired open surgically (5.1%). The 34 procedures, including vascular access, embolization technique, and target vessels of interventions are listed in Supplemental Table 2.

### Secondary EL1 Following pEL2

Four patients in the pEL2 group developed secondary EL1 (8.9%). The median time from initial EL2 detection to EL1 detection was 1278 days (662–2121). Two of the four patients were converted to open surgery for concomitant EL1a and EL1b. Naturally, the pEL2 was resolved by this treatment. One other patient was treated with implantation of a proximal extension stent graft and endoanchors. Postoperative CTA demonstrated pEL2 and successful EL1a exclusion. The AAA remained stable and duplex sonography did not show a pEL2 441 days following reintervention for EL1. The other patient was treated with proximal stent graft extension and implantation of endoanchors for EL1a. As the EL1a persisted, the patient was subsequently treated with double-chimney EVAR for the superior mesenteric artery and the left renal artery, which successfully excluded the EL1a. The EL2 persisted during FU and was treated successfully through transbrachial embolization, using onyx for continued AAA expansion at postoperative day 3079. Patients with pEL2 and secondary EL1 are summarized in Table 3.

**Table 3.** Patients With pEL2 and Secondary EL1: Time From pEL2 Detection to EL1 Detection in Days, Secondary EL1 Type, and Procedures Used to Treat the EL1 (Time Since Index Procedure in Days).

ID	Time from pEL2 detection to EL1 detection	EL1 type	Procedures
181	854	EL1a and EL1b	Open surgical conversion (964)
240	1702	EL1a	- Balloon dilatation of proximal anchor zone and implantation of endoanchors (1728) - Proximal stent graft extension, double-chimney grafts for the SMA, and LRA (1914) - Transbrachial embolization of pEL2 (Onyx; 3079)
248	662	EL1a	Proximal stent graft extension and implantation of endoanchors (1267)
284	2121	EL1a & EL1b	Open surgical conversion (2152)

Abbreviations: EL1, type 1 endoleak; LRA, left renal artery; pEL2, persisting type 2 endoleak; SMA, superior mesenteric artery.

## Discussion

The incidence of EL2 following EVAR ranges between 20% and 40% of cases.^7,11–15^ Most EL2s can be classified as early and transient with only limited clinical relevance. However, persisting EL2s remain a challenge in decision-making during FU as the associated long-term risks are not easily determined. The pEL2 are known to be associated with AAA expansion in about 70% of cases after 5 years of FU, which can be interpreted as a surrogate for continued risk of rupture.^12,13,21–24^ Interestingly, although AAA expansion has been reported frequently in the presence of pEL2, increased rates of secondary ruptures and increased AAA-related mortality are scarcely reported in the literature.^24,25,13^ To further complicate the decision-making process in clinical practice, reinterventions for EL2 can be technically challenging with only moderate clinical success rates on the patients’ side and a financial burden on the public health side.^26,27^ Within this context, the optimal management strategy of pEL2 is still subject to debate 30 years after the introduction of EVAR in 1991.^1^

This study aimed to determine the risk for AAA expansion in patients with pEL2, processing the data of 773 EVAR cases in a single center during a 15-year period. Persisting EL2 (persisting >12 months in CTA) were detected in 5.8% of all EVAR cases and a relevant AAA expansion of ≥5 mm was observed in 33.3% during a mean FU of 32 months. According to Kaplan–Meier estimates, 45.7% (19.2%–63.6%) of patients with pEL2 will show AAA expansion ≥5 mm after 4 years of FU. Conversely, only 20.9% (0%–40.2%) will have demonstrated relevant AAA shrinkage at 4 years of FU. These results are in line with previous studies and demonstrate a well-selected patient cohort, with strict thresholds for the declaration of AAA expansion and according to interobserver-validated measurements that pEL2 is associated with AAA expansion over time.^4,5,12,13,21,22^ To address confounding factors that influence AAA expansion following EVAR, the groups under investigation in this study were matched for age and gender, which are two known factors that influence the rate of sac expansion. Furthermore, the groups were also comparable in key anatomic criteria, namely, AAA neck morphology and AAA diameter, which also represent factors that are known to be associated with a higher risk of AAA expansion.^5,10^ Furthermore, this study also demonstrated secondary, concomitant EL1 in 4 out of 45 patients with pEL2 during midterm FU (8.9%), which has been indicated in other recent studies.^28^ These observations of late EL1 in patients with pEL2 might justify further exploration on larger cohorts of patients; however, with the limited number of cases in this study, these findings must be interpreted cautiously as the observed incidence of EL1 might as well be the consequence of natural progression of disease, independent of the presence of a persisting EL2.

Many studies have attempted to predict pEL2.^21,29–33^ Although a deterministic prediction of pEL2 seems to be difficult and pEL2 remains enigmatic in many cases, this study supports the evidence that EL2 persistency is associated with the absolute number of infrarenal aortic branches as patients in the pEL2 group had a significantly increased number of LA and higher proportions of patients with a patent IMA and MSA, compared with the control group with no endoleak. Interestingly, the previously described association of the incidence of pEL2 with the diameter of the IMA or LAs could not be reproduced in this study.^34^

Once observed, treatment of pEL2 is technically challenging and associated with only moderate clinical success rates. In this study, the clinical success rate was 50.0% and 9 out of 18 patients underwent at least 2 reinterventions to treat the pEL2. These results are comparable to the reported success rates in the literature.^26,27^ Furthermore, this study demonstrates, by example, that recurrent reinterventions for pEL2 harbor potential risks for considerable morbidity. Two patients (11.1%) suffered from ischemia of the lumbosacral plexus. In one patient, symptoms resolved without sequelae, the other patient, however, remained wheelchair-bound during FU. Exemplary reports on this devastating complication following embolization of EL2 can be found in the literature as well.^35,36^

In the light of the high rate of AAA expansion and its potential association with adverse late outcomes, as well as the moderate clinical success rates of reinterventions for pEL2 and the associated morbidity, the risk of pEL2 should be considered when the indication for EVAR is made. However, whereas AAA expansion due to EL2 and associated late adverse events have been reported in the literature, results from this study and other studies did not show a significant difference in overall survival or AAA-related mortality when comparing patients with pEL2 with a group of patients with no EL. This disparity demonstrates the necessity of further studies to clarify the association between AAA expansion in the presence of pEL2 with late adverse events.^2,28,37–39^

With regard to the control group, no patient in the control group showed AAA expansion in the absence of any EL. After a mean FU of 32 months, 71.1% of patients demonstrated relevant (≥5 mm) AAA shrinkage. Continued absence of EL in CTA after 1 year of FU advocates for sonographic- and plane *x*-ray surveillance to reduce radiation and contrast volume. After 4 years of FU, according to Kaplan–Meier estimates, only 19.3% (9.2%–40.8%) of patients did not show relevant AAA shrinkage ≥5 mm.

A notable limitation of the study is the small sample size. Due to the limited number of cases, the pEL2 group and the control group were only matched for age and gender. Furthermore, although analysis of key aneurysm morphology characteristics, such as AAA diameter, AAA neck length, AAA neck diameter, and AAA neck angulation did not show statistically significant differences, uncontrolled confounding factors need to be considered as a limitation of the study.

Another limitation of the study is incompleteness of FU, which is a well-known limitation of retrospective study designs and increases the risk of bias. Furthermore, duplex sonography was not considered, which might also have led to selection bias with regard to the actual number of patients who develop pEL2. However, for the retrospective study design of this study, CTA was considered the most adequate tool to diagnose EL2, to verify its persistency, and to reliably measure AAA diameter over time during FU. In addition, CTA allowed interobserver and intraobserver validation with retrospective measurements. In spite of these limitations, this study still represents one of the larger series of patients with pEL2 reporting on AAA sac evolution from a single center over a period of 15 years.

## Conclusions

The diagnosis of pEL2 suggests yearly FU surveillance with reliable AAA diameter monitoring to identify candidates for reintervention. Further studies are warranted to evaluate the association of AAA expansion due to pEL2 with clinical outcomes to allow recommendations with regard to treatment indications.

## Supplemental Material

sj-docx-1-jet-10.1177_15266028221081079 – Supplemental material for Persisting Type 2 Endoleaks Following EVAR for AAA Are Associated With AAA Expansion Click here for additional data file. Supplemental material, sj-docx-1-jet-10.1177_15266028221081079 for Persisting Type 2 Endoleaks Following EVAR for AAA Are Associated With AAA Expansion by Johannes Hatzl, Vivian Wang, Maani Hakimi, Christian Uhl, Fabian Rengier, Thomas Bruckner and Dittmar Böckler in Journal of Endovascular Therapy

sj-docx-2-jet-10.1177_15266028221081079 – Supplemental material for Persisting Type 2 Endoleaks Following EVAR for AAA Are Associated With AAA Expansion Click here for additional data file. Supplemental material, sj-docx-2-jet-10.1177_15266028221081079 for Persisting Type 2 Endoleaks Following EVAR for AAA Are Associated With AAA Expansion by Johannes Hatzl, Vivian Wang, Maani Hakimi, Christian Uhl, Fabian Rengier, Thomas Bruckner and Dittmar Böckler in Journal of Endovascular Therapy

sj-tiff-3-jet-10.1177_15266028221081079 – Supplemental material for Persisting Type 2 Endoleaks Following EVAR for AAA Are Associated With AAA Expansion Click here for additional data file. Supplemental material, sj-tiff-3-jet-10.1177_15266028221081079 for Persisting Type 2 Endoleaks Following EVAR for AAA Are Associated With AAA Expansion by Johannes Hatzl, Vivian Wang, Maani Hakimi, Christian Uhl, Fabian Rengier, Thomas Bruckner and Dittmar Böckler in Journal of Endovascular Therapy

sj-tiff-4-jet-10.1177_15266028221081079 – Supplemental material for Persisting Type 2 Endoleaks Following EVAR for AAA Are Associated With AAA Expansion Click here for additional data file. Supplemental material, sj-tiff-4-jet-10.1177_15266028221081079 for Persisting Type 2 Endoleaks Following EVAR for AAA Are Associated With AAA Expansion by Johannes Hatzl, Vivian Wang, Maani Hakimi, Christian Uhl, Fabian Rengier, Thomas Bruckner and Dittmar Böckler in Journal of Endovascular Therapy

sj-tiff-5-jet-10.1177_15266028221081079 – Supplemental material for Persisting Type 2 Endoleaks Following EVAR for AAA Are Associated With AAA Expansion Click here for additional data file. Supplemental material, sj-tiff-5-jet-10.1177_15266028221081079 for Persisting Type 2 Endoleaks Following EVAR for AAA Are Associated With AAA Expansion by Johannes Hatzl, Vivian Wang, Maani Hakimi, Christian Uhl, Fabian Rengier, Thomas Bruckner and Dittmar Böckler in Journal of Endovascular Therapy

sj-tiff-6-jet-10.1177_15266028221081079 – Supplemental material for Persisting Type 2 Endoleaks Following EVAR for AAA Are Associated With AAA Expansion Click here for additional data file. Supplemental material, sj-tiff-6-jet-10.1177_15266028221081079 for Persisting Type 2 Endoleaks Following EVAR for AAA Are Associated With AAA Expansion by Johannes Hatzl, Vivian Wang, Maani Hakimi, Christian Uhl, Fabian Rengier, Thomas Bruckner and Dittmar Böckler in Journal of Endovascular Therapy

sj-tiff-7-jet-10.1177_15266028221081079 – Supplemental material for Persisting Type 2 Endoleaks Following EVAR for AAA Are Associated With AAA Expansion Click here for additional data file. Supplemental material, sj-tiff-7-jet-10.1177_15266028221081079 for Persisting Type 2 Endoleaks Following EVAR for AAA Are Associated With AAA Expansion by Johannes Hatzl, Vivian Wang, Maani Hakimi, Christian Uhl, Fabian Rengier, Thomas Bruckner and Dittmar Böckler in Journal of Endovascular Therapy

sj-tiff-8-jet-10.1177_15266028221081079 – Supplemental material for Persisting Type 2 Endoleaks Following EVAR for AAA Are Associated With AAA Expansion Click here for additional data file. Supplemental material, sj-tiff-8-jet-10.1177_15266028221081079 for Persisting Type 2 Endoleaks Following EVAR for AAA Are Associated With AAA Expansion by Johannes Hatzl, Vivian Wang, Maani Hakimi, Christian Uhl, Fabian Rengier, Thomas Bruckner and Dittmar Böckler in Journal of Endovascular Therapy
