# Supplementary material for: Persisting Type 2 Endoleaks Following EVAR for AAA Are Associated With AAA Expansion
Source: J Endovasc Ther. 2022 Mar 3;30(3):372–81. doi: 10.1177/15266028221081079 (PMC10209501; doi:10.1177/15266028221081079)
Supplement: sj-docx-1-jet-10.1177_15266028221081079 – Supplemental material for Persisting Type 2 Endoleaks Following EVAR for AAA Are Associated With AAA Expansion [file sj-docx-1-jet-10.1177_15266028221081079.docx]

| Number of LAs | 0 | 1 | 2 | 3 | 4 | 5 | 6 | 7 | 8 |
| --- | --- | --- | --- | --- | --- | --- | --- | --- | --- |
| Control (N=42) | 6 (14.3%) | 7 (16.7%) | 7  (16.7%) | 5  (11.9%) | 4  (9.5%) | 6  (14.3%) | 5  (11.9%) | 1  (2.4%) | 1  (2.4%) |
| pEL2 (N=40) | 0  (0%) | 1  (2.5%) | 2  (5.0%) | 5  (12.5%) | 13  (32.5%) | 9  (22.5%) | 6  (15.0%) | 3  (7.5%) | 1  (2.5%) |

Supplementary table 1: Distribution of the number of lumbar arteries (LA)

LA: lumbar artery, pEL2: persisting type 2 endoleak (> 12 months)
