# Supplementary material for: Persisting Type 2 Endoleaks Following EVAR for AAA Are Associated With AAA Expansion
Source: J Endovasc Ther. 2022 Mar 3;30(3):372–81. doi: 10.1177/15266028221081079 (PMC10209501; doi:10.1177/15266028221081079)
Supplement: sj-docx-2-jet-10.1177_15266028221081079 – Supplemental material for Persisting Type 2 Endoleaks Following EVAR for AAA Are Associated With AAA Expansion [file sj-docx-2-jet-10.1177_15266028221081079.docx]

Supplementary table 2: Vascular access, embolization technique, target vessels and clinical success of reinterventions for pEL2

| ID | Vascular access | Embolization technique | Feeder vessel(s) | Clinical success |
| --- | --- | --- | --- | --- |
| 200 | Transbrachial | - | - | no |
| 200 | Transfemoral | Coil | IMA | no |
| 200 | Transfemoral | Histoacryl/Lipidol | LA | yes |
| 207 | Transfemoral | - | - | no |
| 207 | Transbrachial | Onyx | IMA | no |
| 207 | Transfemoral | Onyx & Coil | LA | yes |
| 211 | Transfemoral | - | - | no |
| 240 | Transbrachial | Onyx | LA | indetermined |
| 248 | Transfemoral | - | - | no |
| 248 | AAA sac puncture | - | - | no |
| 287 | Transfemoral | Coil | LA | yes |
| 299 | Transfemoral | HA/Lipidol | IMA & LA | no |
| 299 | AAA sac puncture | Coil | IMA & LA | indetermined |
| 352 | Transbrachial | - | - | no |
| 352 | AAA sac puncture | - | - | no |
| 352 | Laparotomy | Surgical ligation | IMA & LA | yes |
| 368 | Transfemoral | HA/Lipidol | LA | yes |
| 370 | Transfemoral | - | - | no |
| 370 | AAA sac puncture | HA/Lipidol & Coil | LA | no |
| 370 | AAA sac puncture | HA/Lipidol & Coil | LA | indetermined |
| 388 | Transfemoral | - | - | no |
| 388 | Transfemoral | - | - | no |
| 388 | AAA sac puncture | Onyx | LA | no |
| 388 | AAA sac puncture | Onyx | LA | no |
| 388 | Transfemoral | - | - | no |
| 515 | Transfemoral | - | - | no |
| 515 | Transfemoral | HA/Lipidol | LA | yes |
| 523 | Laparoscopy | Surgical clipping | IMA | no |
| 523 | Laparoscopy | Surgical clipping | IMA | yes |
| 537 | Transfemoral | - | - | no |
| 634 | Transbrachial | HA/Lipidol & Coil | LA | yes |
| 661 | Transfemoral | - | - | no |
| 670 | Transfemoral | - | - | indetermined |
| 735 | Transfemoral | Onyx | LA | yes |

HA: Histoacryl; LA: lumbar artery; IMA: inferior mesenteric artery
