# Supplementary figures and images for: Persisting Type 2 Endoleaks Following EVAR for AAA Are Associated With AAA Expansion
Source: J Endovasc Ther. 2022 Mar 3;30(3):372–81. doi: 10.1177/15266028221081079 (PMC10209501; doi:10.1177/15266028221081079)

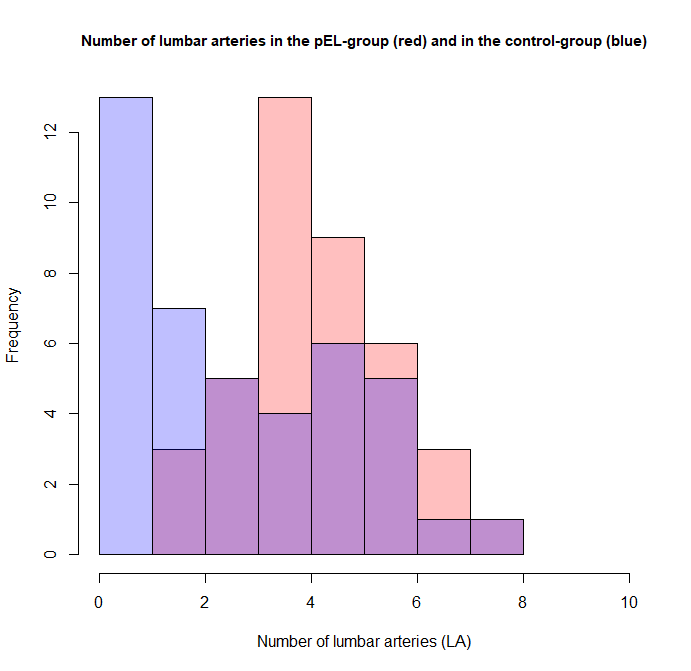

Supplement: sj-tiff-3-jet-10.1177_15266028221081079 – Supplemental material for Persisting Type 2 Endoleaks Following EVAR for AAA Are Associated With AAA Expansion [file sj-tiff-3-jet-10.1177_15266028221081079.tiff]

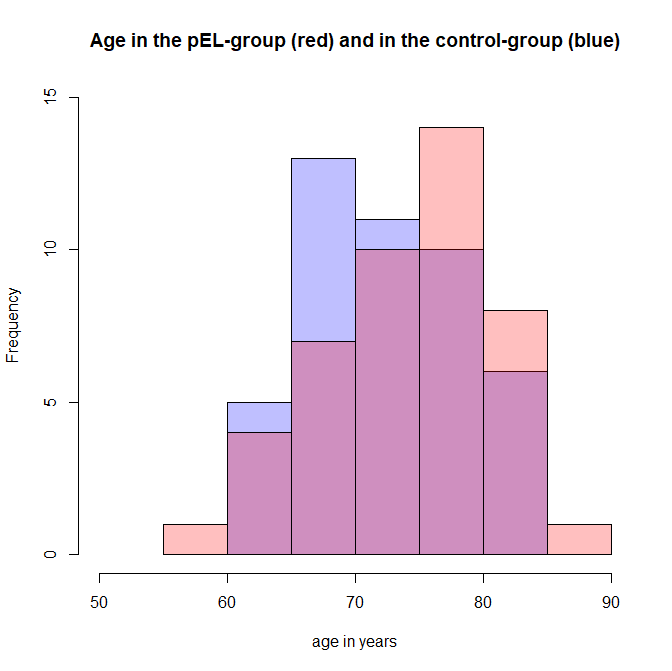

Supplement: sj-tiff-4-jet-10.1177_15266028221081079 – Supplemental material for Persisting Type 2 Endoleaks Following EVAR for AAA Are Associated With AAA Expansion [file sj-tiff-4-jet-10.1177_15266028221081079.tiff]

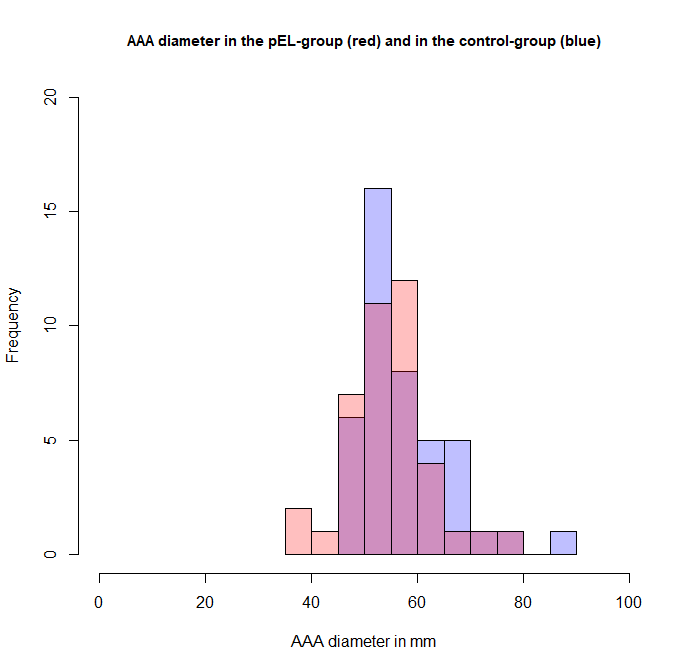

Supplement: sj-tiff-5-jet-10.1177_15266028221081079 – Supplemental material for Persisting Type 2 Endoleaks Following EVAR for AAA Are Associated With AAA Expansion [file sj-tiff-5-jet-10.1177_15266028221081079.tiff]

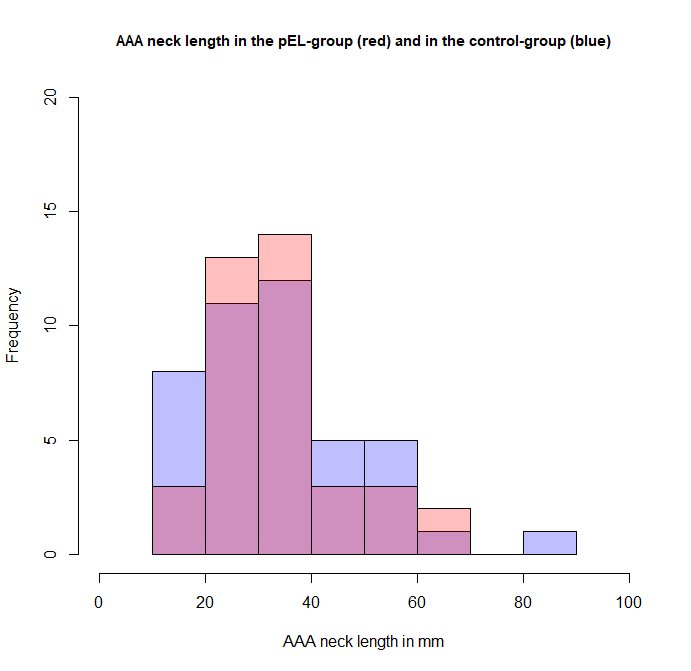

Supplement: sj-tiff-6-jet-10.1177_15266028221081079 – Supplemental material for Persisting Type 2 Endoleaks Following EVAR for AAA Are Associated With AAA Expansion [file sj-tiff-6-jet-10.1177_15266028221081079.tiff]

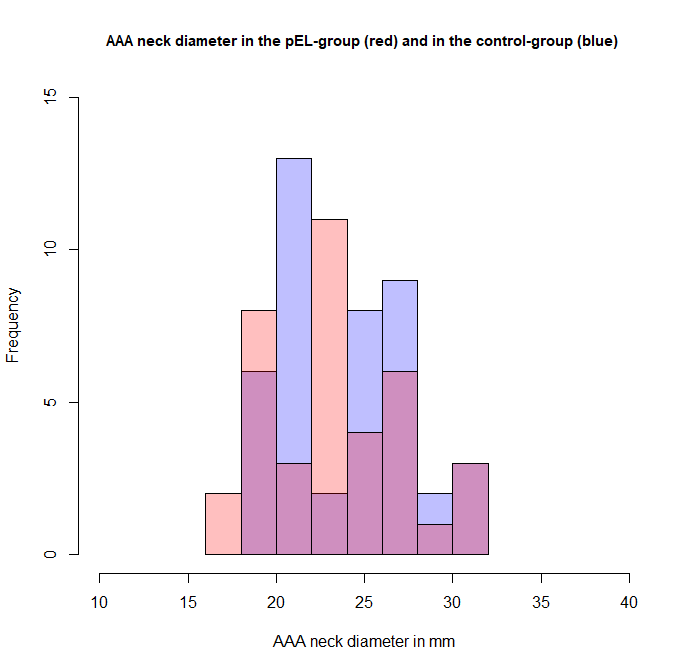

Supplement: sj-tiff-7-jet-10.1177_15266028221081079 – Supplemental material for Persisting Type 2 Endoleaks Following EVAR for AAA Are Associated With AAA Expansion [file sj-tiff-7-jet-10.1177_15266028221081079.tiff]

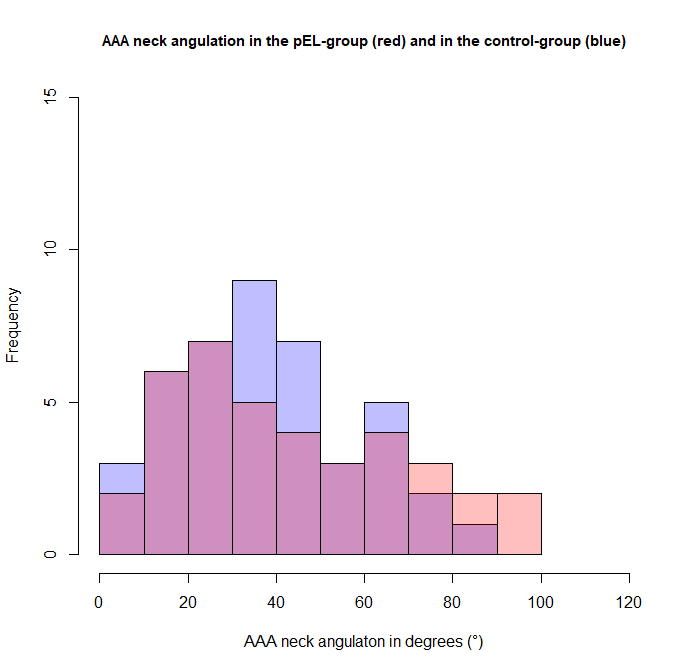

Supplement: sj-tiff-8-jet-10.1177_15266028221081079 – Supplemental material for Persisting Type 2 Endoleaks Following EVAR for AAA Are Associated With AAA Expansion [file sj-tiff-8-jet-10.1177_15266028221081079.tiff]
